# Fabrication of inverse opal molybdenum sulfide and its use as a catalyst for H_2_ evolution†

**DOI:** 10.1039/d3ra02972g

**Published:** 2023-09-20

**Authors:** Thai D. Nguyen, Huong T. L. Phung, Duc N. Nguyen, Anh D. Nguyen, Phong D. Tran

**Affiliations:** a University of Science and Technology of Hanoi, Vietnam Academy of Science and Technology 18 Hoang Quoc Viet Hanoi Vietnam nguyen-duc.anh@usth.edu.vn tran-dinh.phong@usth.edu.vn; b Graduated University of Science and Technology, Vietnam Academy of Science and Technology 18 Hoang Quoc Viet Hanoi Vietnam

## Abstract

Amorphous molybdenum sulfide (MoS_*x*_) and crystalline molybdenum disulfide (MoS_2_) are attractive noble-metal-free electrocatalysts for the H_2_ evolution reaction from water. Their actual activities depend on the quantity of active sites which are exposed to the electrolyte, which in turn, is influenced by their specific electrochemical surface area. Herein we report on the fabrication of regular inverse opal MoS_*x*_ and MoS_2_ films by employing polystyrene nanoparticles with diameters in the range of 30–90 nm as hard templates. The use of these catalysts for the H_2_ evolution reaction in an acidic electrolyte solution is also presented. Impacts of the regular porous structure, the film thickness as well as the chemical nature of the catalyst (MoS_2_*versus* MoS_*x*_) are discussed. It shows a catalytically-effective-thickness of *ca.* 300 nm where the electrolyte can fully penetrate the catalyst macropores, thus all the catalytic active sites can be exposed to the electrolyte to achieve the maximal catalytic operation.

## Abstract 2

Schematical illustration on the HER operation of an inverse opal MoS_*x*_ catalyst when the generated H_2_ limits the catalytic surface active area.

## Graphical abstract

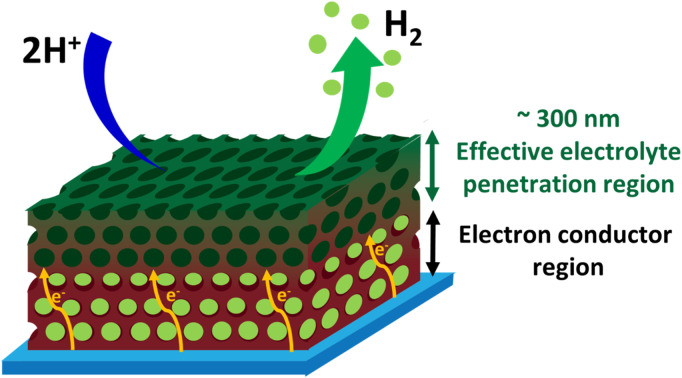

## Introduction

Water splitting is an attractive technology for the large-scale production of green H_2_.^1^ It consists of two half reactions, namely the hydrogen evolution reaction (2H^+^ + 2e^−^ → H_2_) and the oxygen evolution reaction (2H_2_O → O_2_ + 4H^+^ + 4e^−^). Because these reactions require multiple electrons and multiple protons, they have slow kinetics. Between the two, the hydrogen evolution reaction (HER) has faster kinetics in comparison to the oxygen evolution reaction (OER).^2^ Nevertheless, an efficient electrocatalyst is still indispensable to realize large scale H_2_ production with moderate overpotentials.^3^ A prime candidate for this role is platinum (Pt), which requires almost zero overpotential to operate in an acidic electrolyte solution.^4^ However, the rarity of Pt on the Earth's crust hinders its application in industry, where large-scale production of H_2_ is demanded. Thus, great efforts have being mobilized to search for alternative catalysts made of Earth-abundant-elements to replace Pt-based ones.^4*b*,5^ To date, the amorphous non-stoichiometric molybdenum sulfide (denoted hereafter as MoS_*x*_) and the crystalline molybdenum disulfide (denote hereafter as MoS_2_) are known to be among the most promising alternatives to Pt. Substantial information on the H_2_ evolution mechanism on MoS_*x*_ (ref. 5*d* and 6) and MoS_2_ (ref. 4*b* and 7) has been obtained, promoting the identification of appropriate strategies for further boosting the performance of these catalysts. Since the H_2_ evolution reaction occurs on the Mo center, creating a S-vacancy is a valuable strategy to boost the catalytic activity of MoS_2_. It could be realized either by electrochemical oxidation^8^ or plasma treatment.^9^ Introducing another transition metal such as Co is also an efficient strategy to activate the S^2−^ or (S–S)^2−^ centers, which possess almost negligible catalytic activity, thus promoting the overall performance of MoS_2_ and MoS_*x*_ catalysts.^10^ As nanocatalysts, the catalytic activities of MoS_2_ and MoS_*x*_ could be tuned by varying their particle shape and size.^11^ In principle, the smaller the particle is, the larger the specific surface area will be, thus higher population of active centers on the particle's surface will be available and exposed to the electrolyte. As the result, higher catalytic activity could be expected. Indeed, it was demonstrated that by decreasing the size of the MoS_2_ crystals, the density of active sites located on the MoS_2_ edges increased, leading to a significant boost in catalytic activity.^11*a*,*b*^ Nevertherless, it is challenging to fabricate small nanoparticles, *e.g.* having diameter of less than 10 nm, with a narrow size distribution. Moreover, agglomeration of small nanoparticles when they are deposited onto the electrode surface for catalysis operation is a serious problem leading to reduction of the specific surface area.

In this context, the engineering of microporous regular three-dimensional (3D) catalyst appeares to be an attractive strategy to maximize the catalyst's specific electrochemical surface area, thus maximizing the population of active sites exposed to the electrolyte. Through this process, the catalytic activity of the material can be boosted without negative impact to the robustness. In general, the preparation of regular 3D nanomaterials can be classified into soft-templating, hard-templating and template-free preparation methods.^12^ The template-free and soft-templating methods are normally lack of controllability and uniformity, thus are not appropriate for engineering a stable and uniform structure.^12*a*^ On the other hand, the hard-templating method is rather technically complicated due to the strict requirement of a post-treatment step namely the removal of the hard template such as silica or polymers. In compensation for the complicated processing, the hard-templating method could offer well-ordered structures.^13^ Using the hard-templating method, Kibsgaard *et al.*^14^ presented a pioneer work fabricating mesoporous MoS_2_ with a double-gyroid morphology using a double-gyroid silica hard template. Therein, the Mo metal which had been electrodeposited within the silica host underwent sulfidation using H_2_S gas, turning into MoS_2_.^14^ The silica was chemically etched out by a HF solution, generating the 3D-MoS_2_. In a 0.5 M H_2_SO_4_ electrolyte solution, the 3D-MoS_2_ catalyst generated a benchmarking catalytic current density of 10 mA cm^−2^ at an applied potential of −280 mV *versus* the Reversible Hydrogen Electrode (RHE). Deng *et al.* reported on the preparation of MoS_2_ foam using SiO_2_ nanosphere as template, with (NH_4_)_6_Mo_7_O_24_ and CS_2_ as precursors for Mo and S, respectively.^15^ After etching out the SiO_2_ template, MoS_2_ foam nanoparticles having an average pore size of ∼30 nm was obtained. These nanoparticles were then drop-casted onto a glassy carbon electrode using Nafion polymer as the binder to enhance the stability of the catalyst layer. To generate the catalytic current of 10 mA cm^−2^, this MoS_2_-coated glassy carbon electrode required an applied potential of −210 mV *vs.* RHE. Pumera *et al.* demonstrated the use of commercial polystyrene beads (hereafter referred as PS) coated on indium tin oxide (ITO) electrode surface as a template for preparing the microporous amorphous molybdenum sulfide MoS_*x*_.^16^ Therein, the MoS_*x*_ material was fabricated around the template *via* electrodeposition using an (NH_4_)_2_[MoS_4_] deposition bath. When PS particles with diameter of 3 μm or larger was used, only a sub-monolayer of PS particles was obtained, resulting in the deposition of a MoS_*x*_ film with rather low density. Denser MoS_*x*_ film was obtained using multiple layers of PS particles with smaller size, being 0.24 μm in diameter. In an acidic electrolyte, namely 0.5 M H_2_SO_4_ solution, the resultant 3D-MoS_*x*_ required a potential of −240 mV to sustain a catalytic current density of 10 mA cm^−2^. The same approach has been exploited to engineer 3D-MoSe_*x*_ catalysts using a deposition bath constituted of H_2_MoO_4_ and SeO_2_ in 0.2 M NH_4_OH.^17^ It was found that the resultant MoSe_*x*_ was less hydrophobic and thus more catalytically active for the HER when the PS beads used as template are smaller in size.

**Fig. 1:**
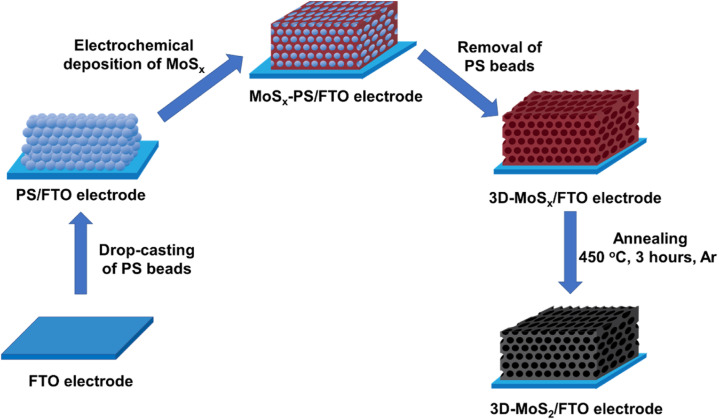
Presentation of the stepwise fabrication process used for the preparation of 3D-MoS_*x*_ and 3D-MoS_2_ films on fluorine-doped tin oxide substrate.

Being inspired by this concept, by using smaller synthetic PS beads template, we aim to fabricate 3D-MoS_*x*_ and 3D-MoS_2_ matrix which have regular structure with higher number of material layers, and thus, higher material density. Fig. 1 schematically illustrates the preparation process that has been developed in this work for preparation of 3D-MoS_*x*_ and 3D-MoS_2_ matrix.

## Experimental methods

### Materials

Potassium persulfate (KPS) (99%), sodium dodecyl sulfate (SDS) (98%), styrene (99%) contained anti-polymerizer *t*-butyl catechol, LiClO_4_ 95%, (NH_4_)_2_MoS_4_ 99.97%, CuSO_4_ 99%, H_2_SO_4_ 98% were purchased from Sigma-Aldrich. Prior to use, the styrene was treated with 10% sodium hydroxide solution in a separatory funnel three times in order to remove the anti-polymerizer.

### Synthesis of spherical polystyrene particles

The polystyrene spherical nanoparticles were synthesized using an emulsion polymerization method described by Yang *et al.*^18^ In a typical experiment, SDS and KPS was dissolved in deionized water in a 100 mL round-bottomed flask equipped with a magnetic stirrer and a rubber septum. A defined amount of styrene was added and the mixture was vigorously stirred for 15 minutes while an N_2_ gas flow was aerated into the emulsionto remove dissolved O_2_. The system was then kept at 70 °C for 24 hours to obtain complete polymerization. The PS nano-beads were collected as suspension and stored at ambient conditions. PS beads with diameter under 100 nm can be fabricated by variation of SDS/styrene weight ratio (Table S1†).

### Synthesis of 3D-MoS_*x*_

The 3D-nanostructured MoS_*x*_ was electrochemically prepared on FTO-coated glass slides using chronoamperometry in aqueous solution with optimized parameters.^19^

#### Preparation of PS/FTO working electrodes

Prior to use, the commercially available FTO sheet was cut into a series of smaller pieces with dimensions of 1 × 2 cm. Electrically insulating thermal tape was used to prevent the connectors of the potentiostat from contacting with the electrolyte, as well as to define an area of 0.283 cm^2^ as the active FTO electrode surface to be used for electrodeposition. The polystyrene beads were deposited on the electrode surface by drop-casting 50 μL of the polystyrene colloidal solution dispersed in water with concentration of 4 mg mL^−1^. The electrode was then dried at room temperature for 3 hours followed by vacuum drying at 10 mbar resulting in a polystyrene coated FTO electrode.

#### Synthesis of 3D-MoS_*x*_

The electrode was then immersed in the (NH_4_)_2_MoS_4_ ^20^ electrolyte solution as the working electrode in a conventional three electrodes setup. Ag/AgCl/KCl 3 M and Pt were used as reference and counter electrodes, respectively. In order to electrochemically grow MoS_*x*_, a potential of 0.4 V *vs.* NHE was applied on the working electrode until the desired amount of electrical charges has been transferred. After rinsing gently with DI water, the 3D-MoS_*x*_ films were finally obtained by the dissolution of the polystyrene beads template by immersing the electrode in dichloromethane for 2 hours. For complete removal of the PS template, this treatment was repeated 5 times with fresh dichloromethane.

### Synthesis of MoS_2_

The previously prepared 3D-MoS_*x*_ was converted into 3D-MoS_2_ by an annealing process under a continuous flow of Ar using a Nabertherm RSH 50/500/13 tube furnace. The temperature ramping rate was kept at 10 °C min^−1^ from room temperature to 450 °C. The furnace was then held at 450 °C for 3 hours and was cooled down naturally to room temperature.

### Materials characterization

Surface morphology analysis and cross-section imaging of the PS/FTO electrodes, MoS_*x*_, 3D-MoS_*x*_ and 3D-MoS_2_ films were performed by a field emission scanning electron microscopy (FE-SEM, Hitachi S4800, Japan) operated at 5 kV. For the SEM cross-section imaging, the back side of the 3D-MoS_*x*_ and 3D-MoS_2_ deposited FTO electrodes were cut by a diamond tip glass cutter following a line going through the middle of the piece. The electrode was then broken into two pieces by applying pressure along the cutting line. SEM images were then collected in different positions on the freshly-exposed edge. Raman spectra were collected by using a LabRAM HR Evolution Raman Microscope (Horiba). Excitation was made by a green 532 nm laser with low power of 0.1 mW to avoid the MoS_*x*_-to-MoS_2_ crystallization.^21^ Size distribution of spherical PS particles have been analyzed employing dynamic laser scattering method using a Horiba SZ-100. Samples were diluted to the concentration of 1 mg mL^−1^ and measured 3 times using 173° back-scatter (NIBS standard), the DLS signals were collected in 120 seconds for each measurement. The water contact angle measurement was assayed using H_2_SO_4_ 0.5 M solution on a Kruss DSA25 drop shape analyzer.

### Electrochemical measurement and catalytic assay

Electrochemical properties and catalytic activities of the MoS_*x*_ and 3D-MoS_*x*_ were assayed in a 0.5 M H_2_SO_4_ (pH 0.6) electrolyte solution. A conventional three electrode configuration was used with MoS_*x*_ coated FTO as the working electrode, Ag/AgCl/3 M KCl as the reference electrode and a Pt wire as the counter electrode. Prior to measurement, the electrolyte solution was purged with N_2_ for 30 minutes to remove dissolved dioxygen. Linear sweep voltammetry analysis was conducted using a Bio-Logic SP-300 potentiostat in the potential range from 0 to −0.4 V *vs.* RHE with a slow potential scan rate of 5 mV s^−1^.

EIS analysis of the samples was assayed in 0.5 M H_2_SO_4_ (pH 0.6) electrolyte solution. Prior to EIS, the samples was assayed for 10 LSV scans from 0 to −0.4 V *vs.* RHE to reach the steady state of catalytic activity. The EIS was conducted at applied potential of −0.3 V *vs.* RHE over the frequency range from 100 000 kHz to 100 000 MHz, the AC probe amplitude was 10 mV.

## Results and discussions

### Fabrication of 3D-MoS_*x*_ material

To the best of our knowledge, the commercially available PS nanoparticles are rather big, *e.g.* with average diameter of few hundreds of nanometers at the very least. Using big PS nanoparticle template would lead to a large volume of space within the catalyst film when the PS template is removed, thus resulting in a low catalyst loading. Herein, aiming to maximize the catalyst loading, we decided to prepare smaller PS nanoparticles with an average diameter ranging from 35 to 90 nm. To this end, the emulsion polymerization of styrene in water with the presence of potassium persulfate (KPS) initiator and sodium dodecyl sulfate (SDS) surfactant was employed (see ESI† for more details). We varied the SDS concentration in pure water to tune the average PS particle size (Table S1†).^22^ Indeed, when the SDS concentration increased, the size of the initial micelles in the emulsion system increased, resulting in formation of larger PS particles.^23^ In all cases, PS nanoparticles with narrow size distribution were obtained (Fig. S1†). These PS nanoparticles were stored in water as stable stock solutions until they were used for the fabrication of the PS nanoparticles template.

**Fig. 2:**
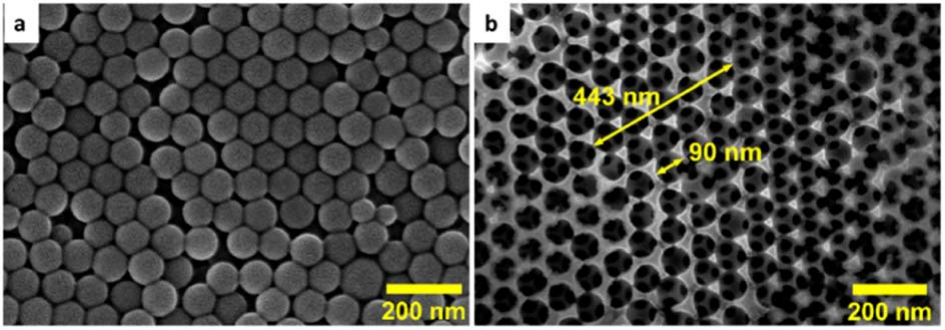
SEM images collected on the surface of (a) a PS (90 nm)–FTO template and (b) a 3D-MoS_*x*_–FTO films after removing the PS particles.

Subsequently, an aliquot volume of a stock solution of PS nanoparticles was drop-casted on a fluorine-doped tin oxide (FTO) substrate surface to obtain a PS–FTO template after the natural evaporation of the solvent in air. The PS–FTO substrate was then immersed in an electrolyte solution constituted of 1 mM (NH_4_)_2_[Mo_3_S_13_] in 0.1 M LiClO_4_ and held at a constant potential of 0.4 V *vs.* NHE. At this potential, [Mo_3_S_13_]^2−^ followed a two-electron oxidation process generating amorphous coordination polymer MoS_*x*_ ^19^ which filled in the space between the PS spherical particles. Immersing the resultant MoS_*x*_–PS–FTO film into dichloromethane solvent induced the dissolution of PS nanoparticles generating the final 3D-MoS_*x*_–FTO film (Fig. 2). We hypothesize that the quality of the 3D-MoS_*x*_–FTO film will depends on (i) the thickness of the PS template, (ii) the size of the PS nanoparticles, and (iii) the amount of MoS_*x*_ deposited.

Theoretical calculation revealed that the 3D-MoS_*x*_ samples possessed significantly superior specific surface areas to that of the bulk MoS_*x*_ film (Table S2†). When comparing PS templates with the same thickness, the one made of smaller PS nanoparticles will provide 3D-MoS_*x*_ with higher specific surface area.

Hence, we first examined the eventual impact of the size of the PS nanoparticles on the quality of the obtained MoS_*x*_ film. The PS nanoparticles having average diameter of 35, 50, 60, 70 and 90 nm were chosen for examination. To this end, the PS particles were re-dispersed into water to obtain a stable colloidal solution having PS weight concentration of 4 mg mL^−1^. An aliquot volume of this colloidal solution was drop-casted onto the surface of a FTO electrode. The solvent was dried naturally to obtain PS–FTO template (Fig. 2a). The PS mass loading was kept at 400 μg cm^−2^ generating an average film thickness of 1650 nm for 90 nm PS particles (Fig. S2†). Fig. S3† shows the surface morphologies of these PS–FTO films. The film with 35 nm PS particles appeared compact with low spacing between the particles. Increasing the particle size generates larger space between the particles. The 3D-MoS_*x*_ film after the removal of 90 nm PS particles shows highly ordered arrangement of MoS_*x*_ in a honeycomb-like structure (Fig. 2b). The spherical space left between the MoS_*x*_ frame was determined to be *ca.* 90 nm in diameter which was exactly the size of the PS nanoparticles used.

**Fig. 3:**
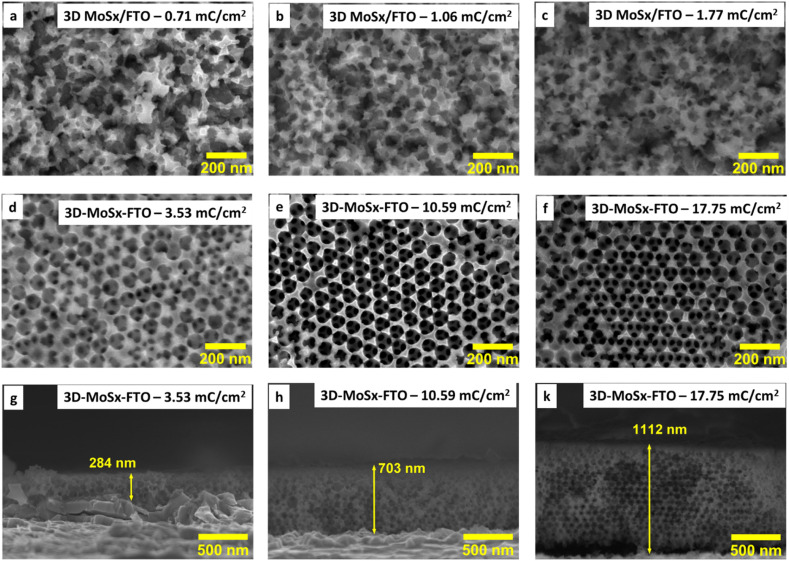
(a)–(f) SEM images collected on the surface of 3D-MoS_*x*_ films grown on FTO substrate using the PS-90 nm breads template but with different deposition charge densities; (g)–(k) SEM cross-section observed for the 3D-MoS_*x*_ films grown with the deposition charge densities of 3.53, 10.59 and 17.65 mC cm^−2^.

We then optimized the growth of MoS_*x*_ on the PS (90 nm)–FTO template by varying the amount of deposition charge. The same oxidation potential of 0.4 V *vs.* RHE and the same deposition bath made of 1 mM (NH_4_)_2_[MoS_4_] in 0.1 M LiClO_4_ electrolyte solution were employed. When the deposition was over, the resultant dark brown films were collected and intensively washed with DI water to remove unwanted species such as LiClO_4_ or (NH_4_)_2_[MoS_4_] that could adsorb on the MoS_*x*_/PS film. The PS particles were finally removed by repeating the immersion of MoS_*x*_–PS–FTO films in dichloromethane to produce the 3D-MoS_*x*_ films on FTO substrate (see ESI† for details). Fig. 3a–f show the surface morphology of the 3D-MoS_*x*_ films after removing the PS nanoparticles template. Using a low deposition charge of 0.71–1.77 mC cm^−2^ resulted in the formation of irregular MoS_*x*_ films where the ordered structure of PS bread templates was not retained (Fig. 3a–c). It is likely because the low deposition charge induced the growth of thin-wall MoS_*x*_ in the space between the PS nanoparticles which were subsequently collapsed when the PS nanoparticle template was dissolved. When the deposition charge of 3.53 mC cm^−2^ was used, more robust MoS_*x*_ wall was deposited which help keep the ordered, honeycomb-like structure of the film intact after the dissolution of the PS nanoparticles (Fig. 3d–f). The cross-section analysis shows clearly the regular three-dimension (3D) structure with a film thickness of 284 nm (Fig. 3g). Indeed, this thickness is equivalent to three layers of PS particles as each particle has an average diameter of 90 nm. This film grew from the FTO surface, indicating a good penetration of [Mo_3_S_13_]^2−^ precursor into the porous PS template. It is also interesting to note that the whole PS particles were successfully removed, including the first layer lying on the FTO surface. In fact, the complete removal of the electrical isolating PS template is critical to ensure good conductivity and thus good electrochemical catalytic activity of the MoS_*x*_ frame.

We then applied the same deposition charge density of 3.53 mC cm^−2^ for other PS–FTO templates. After removing the PS template, the resultant MoS_*x*_ films showed porous but disordered structure if small PS particle size of 35–60 nm was used for the template construction (Fig. S4a–c†). The template with PS particle size of 70 nm resulted in MoS_*x*_ film with a rather ordered structure wherein MoS_*x*_ wall was broken (collapsed) in some location, likely due to its thin and fragile characteristics (Fig. S4d†). Thus, we decided to choose the PS particles of 90 nm in diameter for the construction of PS–FTO template for further optimization of the 3D-MoS_*x*_ fabrication.

**Fig. 4:**
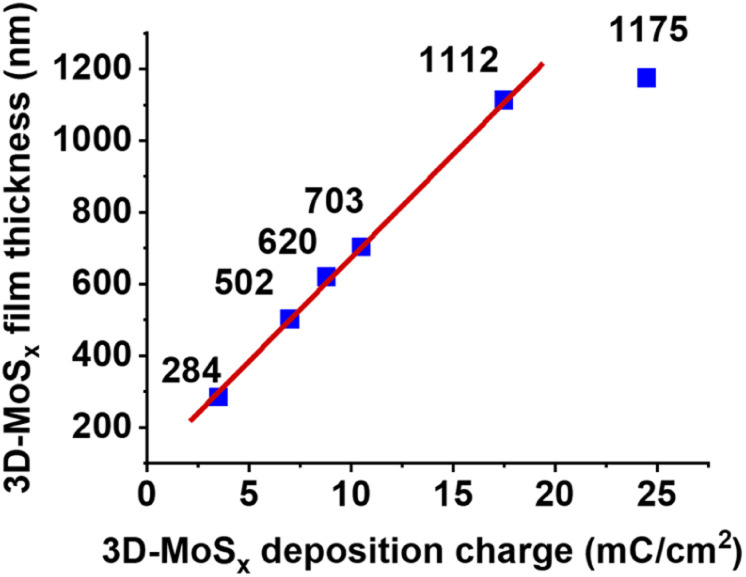
The evolution of 3D-MoS_*x*_ film thickness in function of the deposition charge density consumed.

While holding the same PS-90 nm/FTO template electrode in the deposition bath at the same oxidative deposition potential of 0.4 V *vs.* RHE, we extended the deposition time in order to increase the amount of MoS_*x*_ deposited and thus increase the thickness of the resultant 3D-MoS_*x*_ film. Fig. 3g–k show the cross-section observation of resultant 3D-MoS_*x*_ films having different thickness. Fig. 4 shows the evolution of MoS_*x*_ film thickness in function of the deposition charge consumed. A linear dependence was found for the deposition charge in the range of 3.53–17.85 mC cm^−2^, corresponding to the film thickness range of 284–1112 nm. SEM analysis conducted on the surface of the resultant 3D-MoS_*x*_ films shows regular honeycomb-like pattern (Fig. 3e, f and S5a†). We could obtain even thicker films having thickness of 1175 nm by consuming a deposition charge density of 24.71 mC cm^−2^. However, this film shows irregular surface morphology where both honeycomb-like pattern and compact MoS_*x*_ domains are found (Fig. S5b†). The thickness of 1112 nm corresponds to 12 layers of PS beads of 90 nm diameter. It means that (i) the [Mo_3_S_13_]^2−^ precursor can deeply penetrate into the μm-thick PS films, and (ii) the resultant μm-thick 3D-MoS_*x*_ is robust and remain intact during the dissolution of PS beads.

### Fabrication of 3D-MoS_2_

**Fig. 5:**
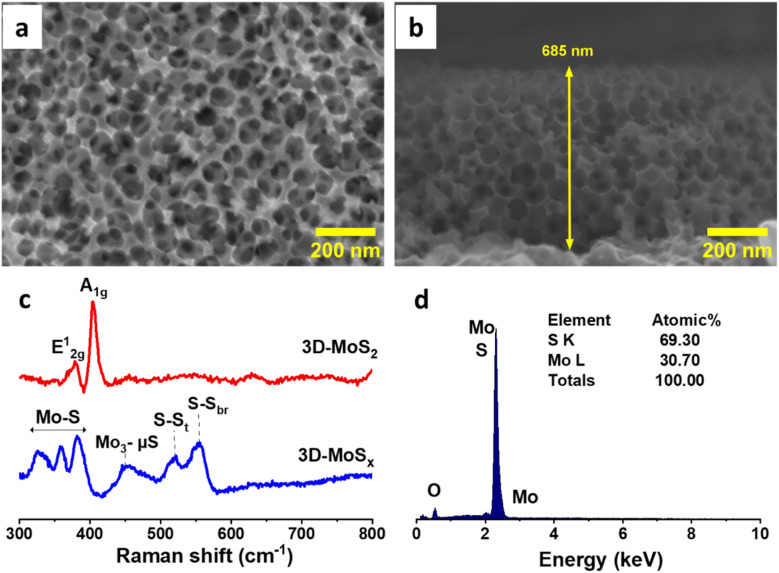
(a) Surface and (b) cross-section SEM images collected on the 3D-MoS_2_ film which was generated from annealing the 3D-MoS_*x*_ film (made with a deposition charge of 10.59 mC cm^−2^); (c) Raman spectra collected on 3D-MoS_2_ and 3D-MoS_*x*_ samples employing a 532 nm laser excitation, (d) EDX analysis conducted on the 3D-MoS_2_ film.

The aforementioned 3D-MoS_*x*_ films were then annealed at 450 °C in an inert atmosphere of Ar flux for 3 hours. Under this thermal treatment, the amorphous MoS_*x*_ transformed into crystalline MoS_2_ releasing sulfur vapor (MoS_*x*(s)_ → MoS_2(s)_ + S_8(g)_).^21^ Raman analysis clearly evidenced the formation of MoS_2_ with two characteristic vibration bands at 380 and 403 cm^−1^ being assignable to the E_1g_ and A_2g_ vibrational modes of MoS_2_ crystalline structure (Fig. 5c).^5*d*^ SEM analysis conducted on the resultant 3D-MoS_2_ film revealed that it conserved the initial structure of the 3D-MoS_*x*_ film. Indeed, the surface of 3D-MoS_2_ film shows the regular honeycomb-like patterns with the spacing volume of *ca.* 90 nm together with the appearance of some damages, probably due to the rapid evaporation of sulfur by-product that weakens the MoS_2_ wall (Fig. 5a). SEM cross-section analysis revealed the similar thickness of the resultant 3D-MoS_2_ (Fig. 5b) and the initial amorphous 3D-MoS_*x*_ film (Fig. 4h). The EDX analysis showed a Mo : S atomic ratio of 1 : 2.3 (Fig. 5d) that was lower in comparison to that of the as-prepared MoS_*x*_ fim, namely Mo : S atomic ratio of 1 : 3 (Fig. S6†).

### Assaying catalytic H_2_ evolution

**Fig. 6:**
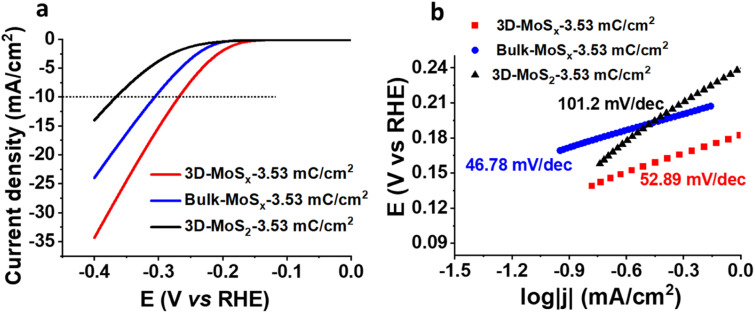
(a) *I*–*V* curves and (b) corresponding Tafel plots recorded for the bulk-MoS_*x*_-3.53 mC cm^−2^, 3D-MoS_*x*_-3.53 mC cm^−2^, and the 3D-MoS_2_-3.53 mC cm^−2^ catalyst electrodes. Electrolyte was a 0.5 M H_2_SO_4_ (pH 0.3) solution. Potential scan rate was 5 mV s^−1^.

We then assayed the H_2_ evolving catalytic activity of 3D-MoS_*x*_ and 3D-MoS_2_ films in a strong acidic electrolyte solution, namely the 0.5 M H_2_SO_4_ (pH 0.3) solution. Prior to measurements, the electrolyte solution was purged with a N_2_ flux to eliminate the dissolved O_2_. In order to examine the potential advantages of the 3D-structuring approach, bulk MoS_*x*_ thin films grown on FTO electrodes without employing the PS template were fabricated and used as reference samples. The deposition charge density was maintained identically to that deployed for making 3D-MoS_*x*_ films to ensure an identical amount of bulk MoS_*x*_ and 3D-MoS_*x*_ grown. Fig. 6a shows *I*–*V* curves recorded on a 3D-MoS_*x*_ electrode and a bulk MoS_*x*_ counterpart, both obtained by employing a deposition charge density of 3.53 mC cm^−2^. Obviously, at any potential the 3D-MoS_*x*_ electrode (red trace) shows higher catalytic current density than that obtained for the bulk MoS_*x*_ electrode (blue trace). To sustain the benchmarking catalytic current density of 10 mA cm^−2^, the 3D-MoS_*x*_-3.53 mC catalyst electrode requires 50 mV less than the bulk-MoS_*x*_-3.53 mC counterpart. The catalytic current density obtained at −0.3 V *vs.* RHE are 15.6 and 9.5 mA cm^−2^ for the 3D-MoS_*x*_-3.53 mC and bulk-MoS_*x*_-3.53 mC electrodes, respectively. Tafel plots in the low kinetic region show comparable slopes, namely 53 mV per decade for the 3D-MoS_*x*_-3.53 mC catalyst electrode and 47 mV per decade for the bulk-MoS_*x*_-3.53 mC catalyst electrode (Fig. 6b). It suggests the similar H_2_ evolution mechanism occurring on these two catalyst electrodes which is expected as these two electrodes are identical in the chemical nature but only different in topology.

Even when displaying the same regular inverse opal structure to that of the amorphous 3D-MoS_*x*_-3.53 mC cm^−2^ electrode, the crystalline 3D-MoS_2_-3.53 mC cm^−2^ electrode showed significantly lower catalytic activity (black trace, Fig. 6a). At −0.3 V *vs.* RHE, the catalytic current densities were 3.85 mA cm^−2^ and 15.6 mA cm^−2^ for the 3D-MoS_2_-3.53 mC cm^−2^ and the 3D-MoS_*x*_-3.53 mC cm^−2^ electrodes, respectively. We note that the 3D-MoS_2_-3.53 mC cm^−2^ electrode was even less active than the bulk-MoS_*x*_-3.53 mC cm^−2^ counterpart (Fig. 6a). It clearly demonstrates that the MoS_*x*_-to-MoS_2_ crystallization causes a significant loss of catalytic ability. The Tafel slope value of 101.2 was deduced for the crystalline 3D-MoS_2_-3.53 mC cm^−2^ which is significantly higher than those obtained for the MoS_*x*_ electrodes (Fig. 6b). This result indicates different HER mechanisms occurring on the MoS_*x*_ and MoS_2_ catalysts. While the Heyrovski and Tafel steps dominate the H_2_ evolution on MoS_*x*_ catalyst, the Volmer step dominates the reaction on 3D-MoS_2_-3.53 mC cm^−2^ counterpart.^24^

**Fig. 7:**
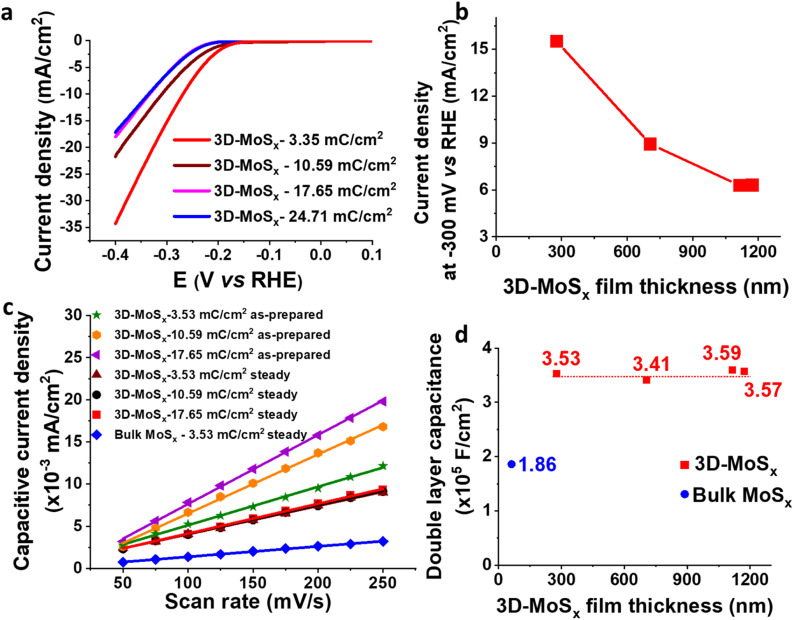
3D-MoS_*x*_ films obtained by using different deposition charge densities, thus having different thickness. (a) *I*–*V* curves, (b) evolution of catalytic current density obtained at −0.3 V *vs.* RHE in function of the 3D-MoS_*x*_ film thickness, (c) capacitive current density recorded at 0.1 V *vs.* RHE in function of potential scan rate for the as-prepared 3D-MoS_*x*_ electrodes and for these electrodes after reaching the steady performance, and (d) evolution of double layer capacitance in function of the 3D-MoS_*x*_ film thickness (catalyst films already reach its steady performance).

We then assayed the stability of 3D-MoS_*x*_-3.53 mC cm^−2^ and 3D-MoS_2_-3.53 mC cm^−2^ catalysts under the catalytic H_2_ evolution conditions in a 0.5 M H_2_SO_4_ electrolyte solution. To this end, the chronopotentiometry analysis was conducted at the benchmarking catalytic current density of 10 mA cm^−2^ for 6 hours. It was found that the 3D-MoS_*x*_-3.53 mC cm^−2^ catalyst required a smaller cathodic potential in comparison to the 3D-MoS_2_-3.53 mC cm^−2^ counterpart to sustain the same catalytic current density (Fig. S7a†). It is consistent with the fact that the 3D-MoS_*x*_-3.53 mC cm^−2^ is more catalytically active than the 3D-MoS_2_-3.53 mC cm^−2^. Interestingly, during the first 10 minutes of operation, potential which was required to apply on the 3D-MoS_*x*_-3.53 mC cm^−2^ catalyst decreased from −0.34 V *vs.* RHE to a steady value of −0.31 V *vs.* RHE (Fig. S8†). It clearly demonstrated an activation stage of amorphous MoS_*x*_ catalyst prior to operation.^6*c*^ When extending the catalytic operation time, higher cathodic potential is required for the 3D-MoS_*x*_-3.53 mC cm^−2^ catalyst suggesting possible degradation (Fig. 7b, red trace). The same tendency was observed for the 3D-MoS_2_-3.53 mC cm^−2^ counterpart in the first 3 hours of operation (Fig. S7b,† black trace). After that, potential suddenly jumped to *ca.* −0.6 V *vs.* RHE (and then more cathodic potential). At this moment, we observed a detachment of 3D-MoS_2_ from the FTO electrode surface. This catalyst detachment could explain for the complete degradation of the catalytic performance.

In order to avoid the catalyst detachment, we conducted the H_2_ evolution assay in a milder condition, namely a chronoamperometry analysis at the constant cathodic potential of −0.3 V *vs.* RHE. The 3D-MoS_2_-3.53 mC cm^−2^ showed a slight superior stability during the operation in comparison to the 3D-MoS_*x*_-3.53 mC cm^−2^. As a demonstration, after 6 h of operation, the 3D-MoS_*x*_-3.53 mC cm^−2^ catalyst electrode lost 73% of its initial catalytic current density (Fig. S7b,† red trace) while the loss was 55% for the 3D-MoS_2_-3.53 mC cm^−2^ one (Fig. S7b,† black trace).

SEM analysis performed on these catalyst electrodes after 6 h-catalysis revealed that the regular inverse opal structure of the 3D-MoS_2_-3.53 mC cm^−2^ catalyst remained whereas the initial structure of 3D-MoS_*x*_-3.53 mC cm^−2^ has been partially corrupted (Fig. S9†).

We then focused on examining the catalytic activities of the 3D-MoS_*x*_ electrodes having different thickness that had been grown by applying different deposition charge density. Fig. 7a shows the *I*–*V* curves recorded on these electrodes. Fig. 7b plots the catalytic current density obtained at potential of −0.3 V *vs.* RHE in function of the film thickness. It can be seen that the 3D-MoS_*x*_-3.53 mC cm^−2^ with the thickness of 284 nm shows the highest catalytic current density. Thicker film shows lower catalytic activity. It demonstrates that the 3D-structuring strategy has its limitation. It could help improving the catalytic activity in comparison to the bulk catalyst film but the effect reaches a plateau after a certain value of film thickness.

### Investigation on the reason behind the limitation of catalytic activity

It is regrettable that micron-meter-thickness 3D-MoS_*x*_ films which possess high density of MoS_*x*_ catalyst could not offer superior catalytic activities as we expected. To understand the reason behind the limitation of catalytic activity, we first measured the surface wettability of the bulk-MoS_*x*_, 3D-MoS_*x*_ and 3D-MoS_2_ films. Water contact angles of 113°, 127° and 128° were determined for the bulk-MoS_*x*_-3.53 mC cm^−2^, 3D-MoS_*x*_-3.53 mC cm^−2^ and 3D-MoS_2_-3.53 mC cm^−2^ films, respectively (Fig. S10†). It means the creation of the inverse opal structure with empty volume where the air could be trapped, *e.g.* sphere diameter of *ca.* 90 nm, insignificantly alters the hydrophobicity of the MoS_*x*_ and MoS_2_ surface. This result indicates that the superiority in catalytic ability of 3D-MoS_*x*_ could be the result of its higher specific electrochemical surface area (ECSA) and/or higher amount of MoS_*x*_ catalyst exposed to the electrolyte but not due to its surface wettability. We determined the relative ECSA of these samples by measuring their double layer capacitance. Fig. S11† shows the *I*–*V* curves recorded on these catalyst electrodes immersed in the same 0.5 M H_2_SO_4_ electrolyte solution in the capacitive potential region, namely 0.05 to 0.15 V *vs.* RHE, at different potential scan rates. Plotting the capacitive current obtained at 0.1 V *vs.* RHE in function of the potential scan rate gives the slope which represents the capacitance (Fig. 7c). The as-prepared 3D-MoS_*x*_-3.53 mC cm^−2^ electrode displayed a double layer capacitance (*C*_dl_) of 4.56 × 10^−5^ F cm^−2^. Increasing the deposition charge, thus increasing the thickness of the 3D-MoS_*x*_ films led to an increment of the double layer capacitance, thus an increment of the ECSA. The capacitance of 7.01 × 10^−5^ F cm^−2^ and 8.19 × 10^−5^ F cm^−2^ were calculated for the 3D-MoS_*x*_-10.59 mC cm^−2^ and the 3D-MoS_*x*_-17.65 mC cm^−2^, respectively (Fig. 7c). Using 1 mM ferrocene (Fc) in dichloromethane as the redox probe (Fig. S12a†), we found that the cathodic current density of the Fc^+^/Fc redox couple increased linearly with the 3D-MoS_*x*_ film's deposition charge (Fig. S12b†) and its thickness (Fig. S12c†). These results clearly demonstrated that increasing the deposition charge resulted in an increment of the electrochemical surface area of 3D-MoS_*x*_ films.

When the 3D-MoS_*x*_-3.53 mC cm^−2^ catalyst electrode already reached its steady catalytic performance for the H_2_ evolution, it displayed a *C*_dl_ of 3.53 × 10^−5^ F cm^−2^ which was slightly smaller than the value of 4.56 × 10^−5^ F cm^−2^ determined for the same electrode before used for catalysis. Under similar conditions, the bulk-MoS_*x*_-3.53 mC cm^−2^ catalyst electrode displayed a *C*_dl_ of 1.86 × 10^−5^ F cm^−2^. It means the 3D-MoS_*x*_-3.53 mC cm^−2^ catalyst has two times higher electrochemical surface area than the bulk-MoS_*x*_-3.53 mC cm^−2^ catalyst (Fig. 7c). Hence, we can attribute the superior catalytic activity of the 3D-MoS_*x*_ catalyst to its superior active surface area in comparison to the bulk-MoS_*x*_-3.53 mC cm^−2^ counterpart. Looking at the 3D-MoS_*x*_-3.53 mC cm^−2^ and the 3D-MoS_2_-3.53 mC cm^−2^, these catalyst electrodes displayed similar capacitance, thus similar specific electrochemical surface area (Fig. S13†).

Interestingly, we found that when the 3D-MoS_*x*_ catalysts reached its steady catalytic performance, it displayed similar capacitance regardless of its thickness, thus similar electrochemical surface areas (Fig. 7c and d). This result suggests that for the thick 3D-MoS_*x*_ films, only a few layers count from the MoS_*x*_/electrolyte interface (thus count from the MoS_*x*_ top surface) are effective for the H_2_ evolution catalysis but not the whole film volume. As mentioned above, the 284 nm-thick 3D-MoS_*x*_-3.53 mC cm^−2^ catalyst film showed the highest catalytic current density. We note that 284 nm is equal to 3-layers of PS nanoparticles, thus 3-layers of MoS_*x*_. Thus, it is likely that only the 3-top-layers of MoS_*x*_ films is catalytically effective for the H_2_ evolution.

To better understand this phenomenon, we investigated the penetration of aqueous H_2_SO_4_ electrolyte in the 3D-MoS_*x*_ electrode volume. To this end, a 707 nm-thick 3D-MoS_*x*_-10.59 mC cm^−2^ electrode was immersed in an electrolyte constituted of 1 mM CuSO_4_ and 0.5 M H_2_SO_4_. A cyclic voltammetry polarization in the potential range of −0.7 to 0.2 V *vs.* Ag/AgCl was then applied to the electrode with a potential scan rate of 50 mV s^−1^ (Fig. S14†). It resulted in the deposition of Cu on the 3D-MoS_*x*_-10.59 mC cm^−2^ electrode. When the deposition was over, the electrode was taken out and then immersed in DI water. The EDS elemental mapping conducted on the cross-section of this electrode showed the homogeneous distribution of Cu, Mo and S elements (Fig. S15†). It clearly demonstrated that the aqueous electrolyte deeply penetrated through all the thickness of the 3D-MoS_*x*_ films.

Hence, prior to the H_2_ evolution catalysis, the electrolyte could penetrate into the whole volume of the 3D-MoS_*x*_ catalyst. However, when the H_2_ evolution occurs during cathodic polarizations, the generated H_2_ bubbles can adsorb on the MoS_*x*_ layers thanks to the H_2_ storage capacity by adsorption of MoS_*x*_.^25^ Thus, the layers being close to the FTO substrate surface are then no longer effective for the H_2_ generation. In other words, only the 3-top-layers of MoS_*x*_ films, where the H_2_ bubbles generated can be evacuated, will be effective for the H_2_ generation.

**Fig. 8:**
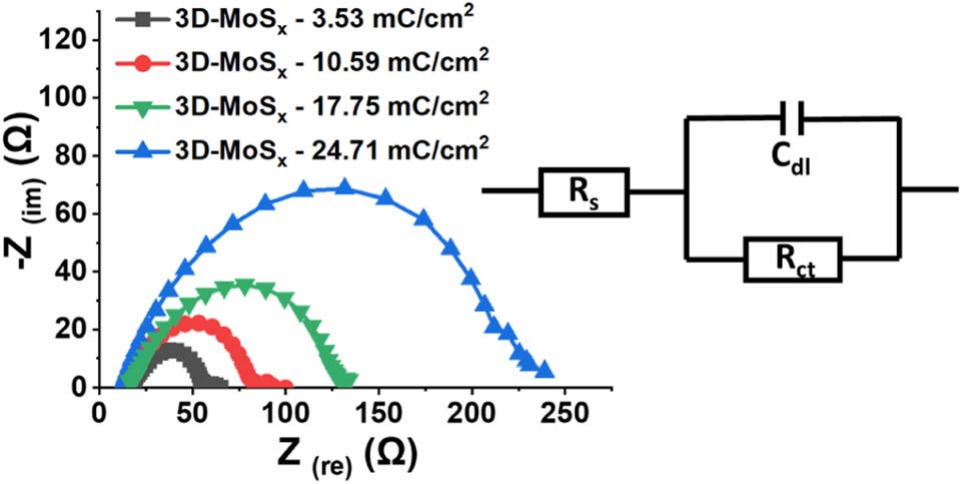
Nyquist plots obtained on 3D-MoS_*x*_ electrodes immersed in a pH 0.3 H_2_SO_4_ electrolyte at −0.4 V *vs.* RHE and equivalent electrical circuit used for fitting the experimental data.

Electrochemical impedance spectroscopic (EIS) analysis is a powerful method to track the electron transfer happening during the H_2_ evolution catalysis. Thus, we conducted the EIS analysis on 3D-MoS_*x*_ electrodes at a constant potential of – 0.3 V *vs.* RHE where the catalytic H_2_ evolution occurred (see ESI† for more details). Fig. 8 shows the obtained Nyquist plots and the simple Randles equivalent electric circuit that we employed to fit the experimental data. *R*_S_ is the external series resistance, *R*_ct_ is the charge transfer resistance at the electrode/electrolyte interface and *C*_dl_ is the capacitance of the 3D-MoS_*x*_ samples. Results obtained from the fitting are presented in Table 1. It can be seen that *R*_ct_ increases drastically with increment of the MoS_*x*_ film thickness. It means a thicker 3D-MoS_*x*_ film has a higher resistance to the electron transfer. In addition, the EIS analyses showed similar capacitance *C*_dl_ values that are in agreement to the similar ECSA values of the 3D-MoS_*x*_ electrodes. The results re-confirmed the limited interfacial area between 3D-MoS_*x*_ electrodes and the electrolyte.

**Table 1:** EIS parameters calculated from simple Randles equivalent circuit

Sample	*R*_s_ (ohm)	*R*_ct_ (ohm)	*C*_dl_ (F)
3D-MoS_*x*_-3.53 mC cm^−2^	19.65	36.45	11.61 × 10^−5^
3D-MoS_*x*_-10.59 mC cm^−2^	17.64	64.86	11.31 × 10^−5^
3D-MoS_*x*_-17.65 mC cm^−2^	18.55	112.3	9.96 × 10^−5^
3D-MoS_*x*_-24.71 mC cm^−2^	12.67	216.4	10.49 × 10^−5^

**Fig. 9:**
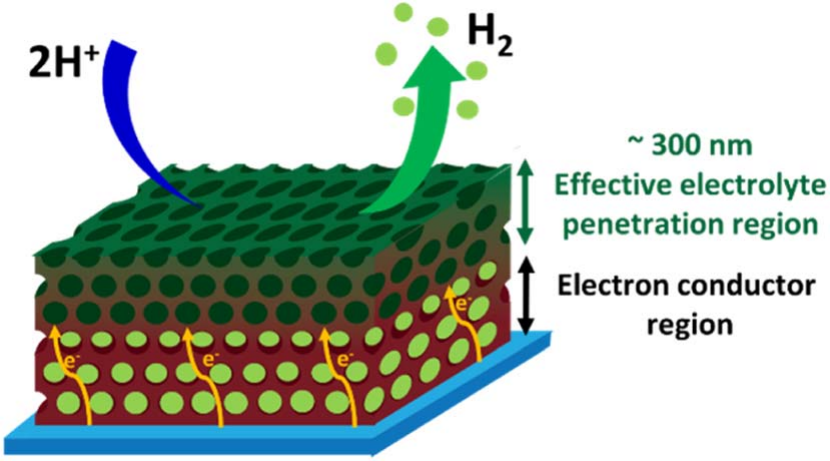
Schematical illustration on the operation of an inverse opal MoS_*x*_ catalyst.

Fig. 9 illustrates schematically the operation of a 3D-MoS_*x*_ or a 3D-MoS_2_ catalyst electrode. The electrolyte could penetrate effectively into the whole volume of the porous catalyst films. When the H_2_ evolution occurs, the H_2_ bubbles generated within the depleted layers of 3D-MoS_*x*_ (or 3D-MoS_2_), *e.g.* the layers stand closely to the FTO substrate surface, could not be effectively evacuated. Thus, the depleted layers of 3D-MoS_*x*_ (or 3D-MoS_2_) will no longer be active for the H_2_ evolution. Therefore, only the few-top-layers of the 3D-MoS_*x*_ (or 3D-MoS_2_) films where H_2_ bubbles can be effectively evacuated will be effective for the H_2_ evolution catalysis. The effective thickness for the H_2_ evolution catalysis in water is about 300 nm, being equivalent to 3-top-layers of 3D-MoS_*x*_ and 3D-MoS_2_. Given that the MoS_*x*_ and MoS_2_ have low electrical conductivities of 10^−4^ to 10^−6^ S cm^−1^,^26^ thicker film displays higher resistance. Consequently, thicker 3D-MoS_*x*_ film shows lower H_2_ evolution catalytic activity. Even having the same effective thickness, and thus the same electrochemical surface area, the 3D-MoS_*x*_ is significantly more active than the 3D-MoS_2_ counterpart. The following reasons could be evoked to explain this phenomenon: (i) the intrinsic activity of an active center within the MoS_2_ is significantly lower than that of an active center within the MoS_*x*_, and/or (ii) the actual number of active sites within MoS_2_ is significantly lower than that within MoS_*x*_ as only the edges of MoS_2_ is active but not its basal plane.^14,27^

## Conclusion

We have successfully fabricated amorphous MoS_*x*_ catalyst electrodes having regular inverse opal structure by using small polystyrene nanoparticles as hard templates. By this fabrication process, we were able to tune the empty space between the MoS_*x*_ wall, thus the density of MoS_*x*_ deposited, by varying the polystyrene particle size. However, using small particles of 30–70 nm diameter size tends to result in 3D-MoS_*x*_ films where the MoS_*x*_ wall is thin and fragile which can be collapsed in some location. Using the template made of 90 nm diameter size polystyrene nanoparticles produces the robust 3D-MoS_*x*_ structure whose thickness can be easily tuned, *e.g.* up to micrometer scale, by extending the MoS_*x*_ electrodeposition time. Annealing the 3D-MoS_*x*_ films at 450 °C under Ar inert atmosphere resulted in the formation of crystalline 3D-MoS_2_ wherein the regular inverse opal structure of the initial MoS_*x*_ film was retained. During catalytic operation, regardless of their actual thickness, the 3D-MoS_*x*_ and 3D-MoS_2_ films display the same effective thickness which is estimated to be *ca.* 284 nm where the water molecule could penetrate and the H_2_ bubbles can be effectively evacuated. Having films thicker than 284 nm is not beneficial and disfavour the H_2_ evolution catalysis as the inner MoS_2_ and MoS_*x*_ layers increase the film resistance. The 3D-MoS_*x*_-284 nm catalyst film shows the highest catalytic activity which is significantly higher than those obtained using the 3D-MoS_2_-284 nm counterpart as well as the bulk-MoS_*x*_ reference film. These results demonstrate that engineering a regular inverse opal structure is a good strategy to enhance the H_2_ evolution catalytic activity of amorphous MoS_*x*_ catalyst, but a fine optimization should be performed to obtain an appropriate thickness, *e.g.* being close to the effective thickness for the penetration of electrolyte.

## Author contributions

Thai D. Nguyen: investigation, writing – original draft; Huong T. L. Phung: investigation; Duc N. Nguyen: methodology, formal analysis; Anh D. Nguyen: conceptualization, methodology, writing-editing/review, project administration, funding acquisition, supervision; Phong D. Tran: conceptualization, writing-editing/review, supervision.

## Conflicts of interest

The authors declare no conflict of interest.

## Supplementary Material

RA-013-D3RA02972G-s001
