# Supplementary material for: Fabrication of inverse opal molybdenum sulfide and its use as a catalyst for H2 evolution
Source: RSC Adv. 2023 Sep 20;13(40):27923–33. doi: 10.1039/d3ra02972g (PMC10510047; doi:10.1039/d3ra02972g)
Supplement: RA-013-D3RA02972G-s001 [file RA-013-D3RA02972G-s001.pdf]

Electronic Supplementary Material (ESI) for RSC Advances.
This journal is © The Royal Society of Chemistry 2023

Supporting information

Fabrication of inverse opal molybdenum sulfide and its use as catalyst for H_2 evolution

Thai D. Nguyen,[a] Huong T. Phung,[a,b] Duc N.Nguyen,[a] Anh D. Nguyen,[a*] Phong D. Tran[a*]

[a]*University of Science and Technology of Hanoi, Vietnam Academy of Science and Technology, 18 Hoang Quoc Viet, Hanoi, Vietnam. Email: nguyen-duc.anh@usth.edu.vn and phong-dinh.tran@usth.edu.vn*

[b]*Graduated University of Science and Technology, Vietnam Academy of Science and Ttechnology, 18 Hoang Quoc Viet, Hanoi, Vietnam*

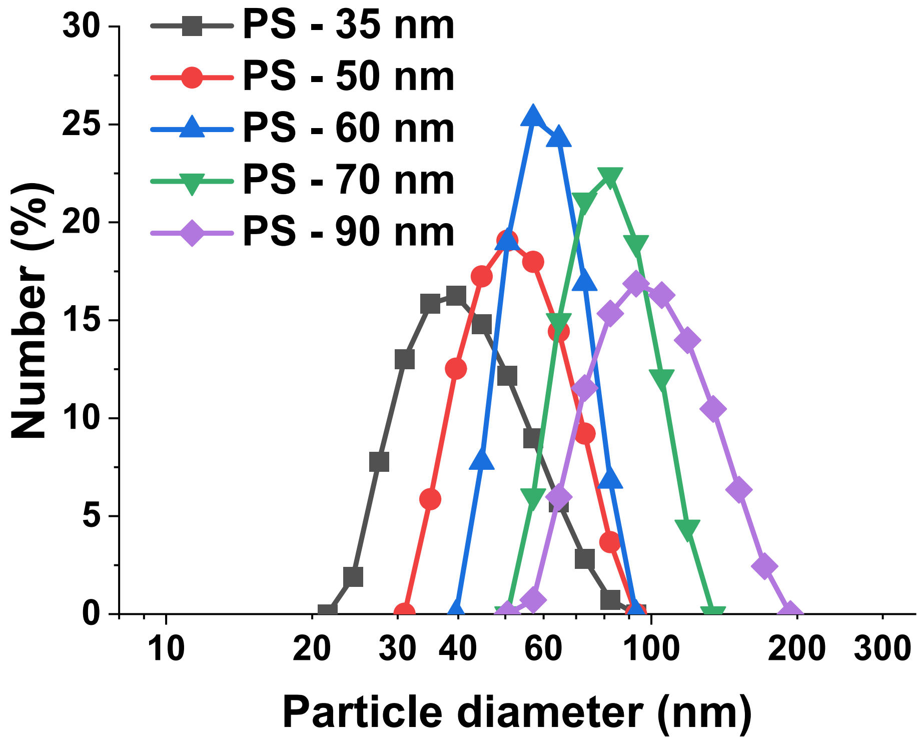

Figure S1. *Size distribution of synthetic spherical polystyrene nanoparticles*

Table S1. Polystyrene particles parameters extracted from SEM and DLS analyses

No.	Styrene (mL)	MeOH/H_2O (mL)	KPS (%w/Sty)	SDS (%w/Sty)	Av. Size (nm) (SEM)	Av. Size (nm) (DLS)	PDI (DLS)
**1**	**1.55**	0/70	2.5	6	**35**	39.4	0.133
**2**	**1.55**	0/70	2.5	4.5	**50**	50.4	0.063
**3**	**1.55**	0/70	2.5	3	**60**	56.0	0.113
**4**	**1.55**	0/70	2.5	2	**70**	75.5	0.077
**5**	**1.55**	0/70	2.5	1.2	**90**	89.2	0.087

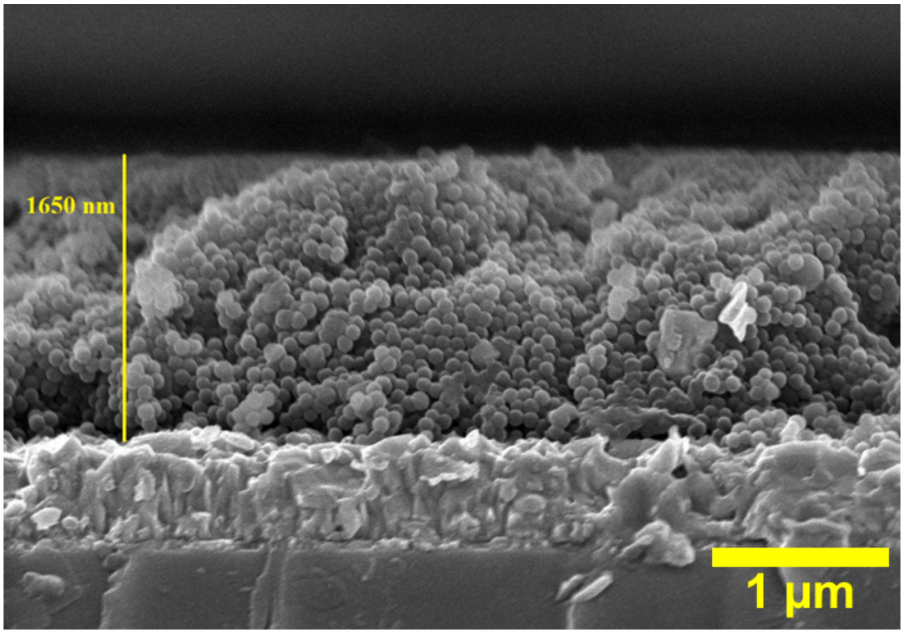

***Figure S2**. SEM cross-section image of PS-90nm beads coated FTO electrode*

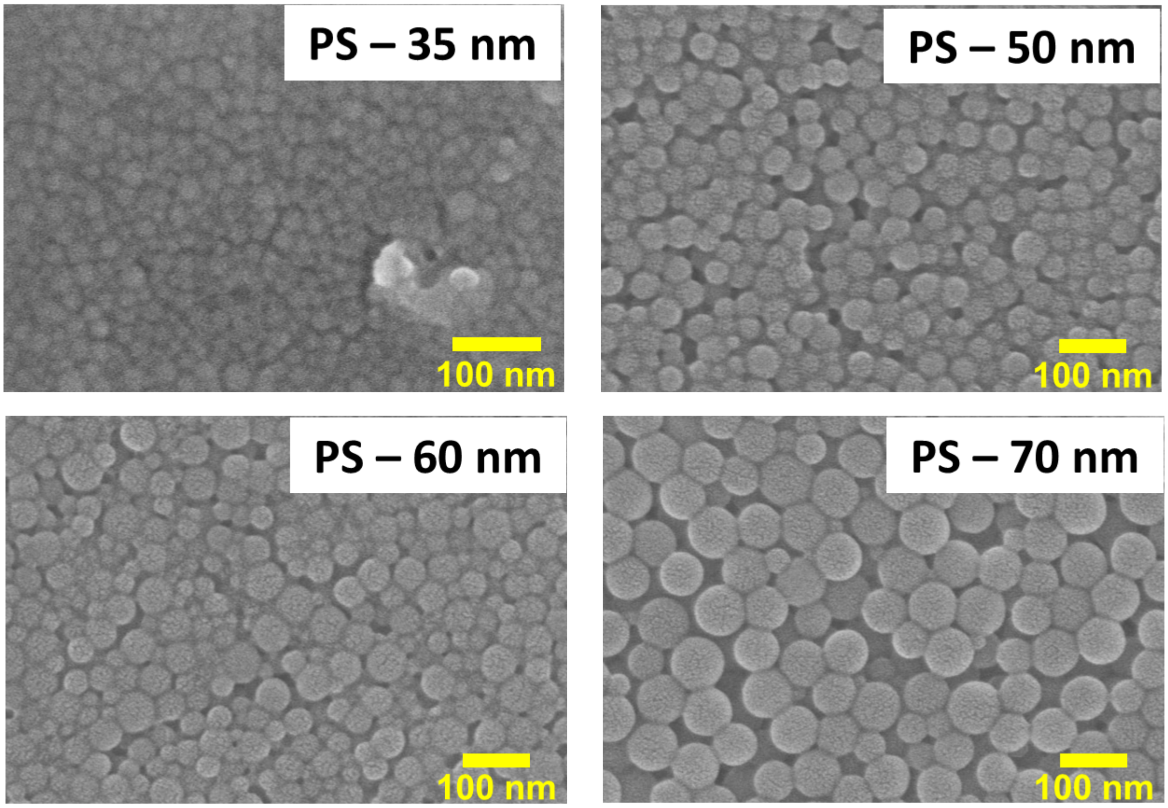

Figure S3. *Surface SEM images of PS-FTO electrodes (using PS particles with different size)*

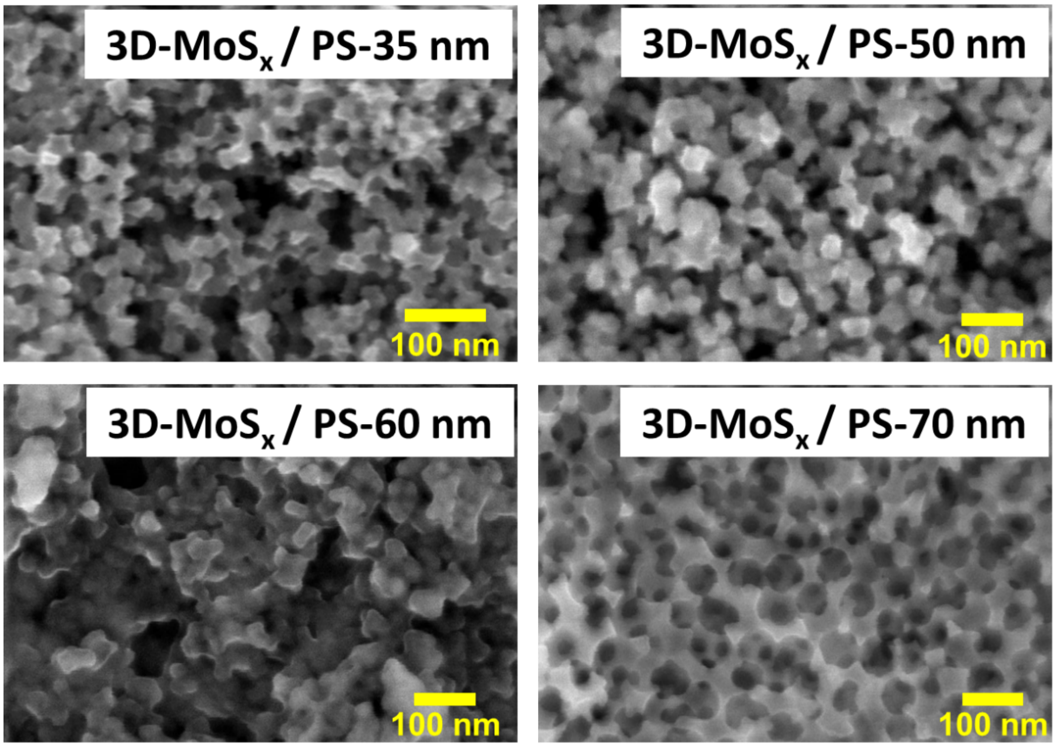

Figure S4. *Surface SEM images of 3D-*MoS_x *films obtained by using 3.53 mC/cm*^{2} *deposition charge and different PS-FTO electrodes (different PS particle size)*

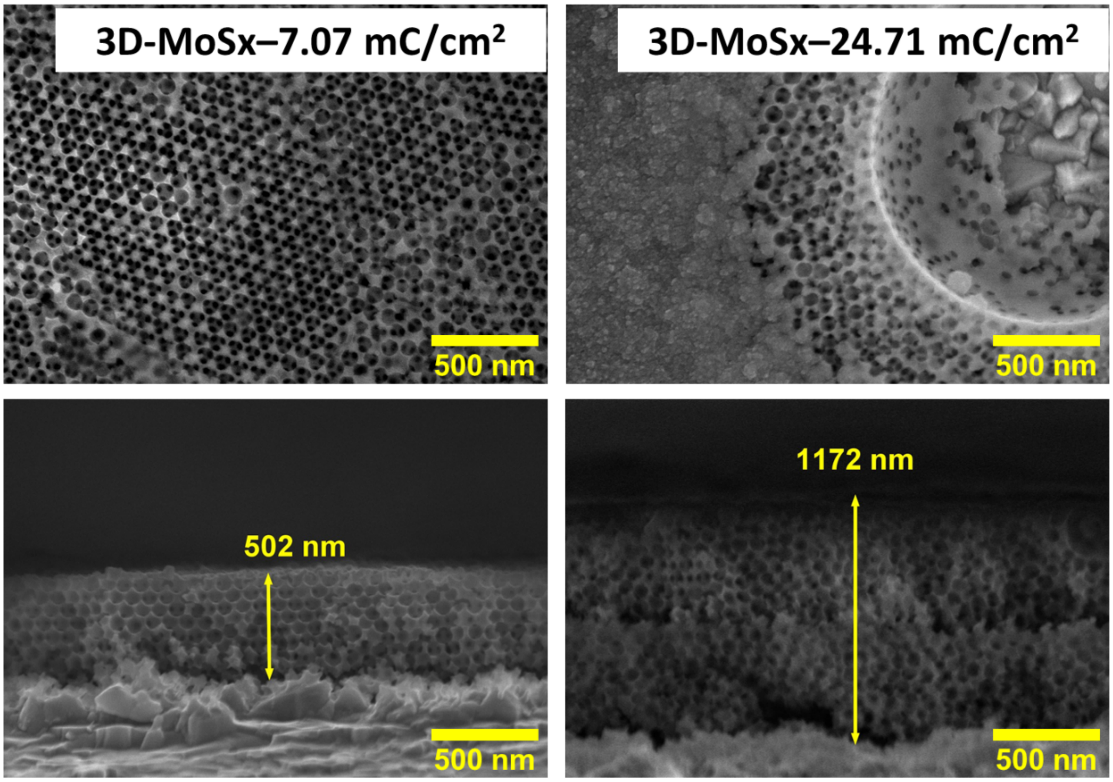

***Figure S5**. Surface and cross-section SEM images collected on 3D-MoS$_x$-7.07 mC/cm^2 and 3D-MoS$_x$-24.71 mC/cm^2*

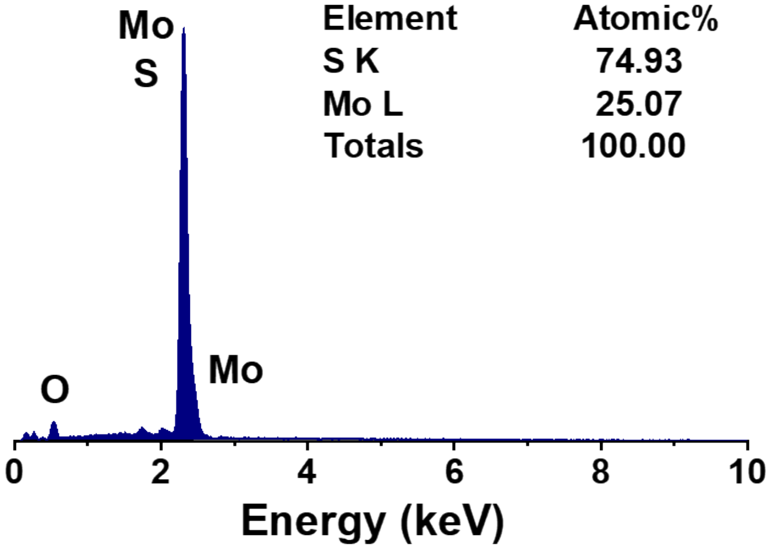

Figure S6. *EDX analysis of an as-prepared 3D-MoS$_x$ film*

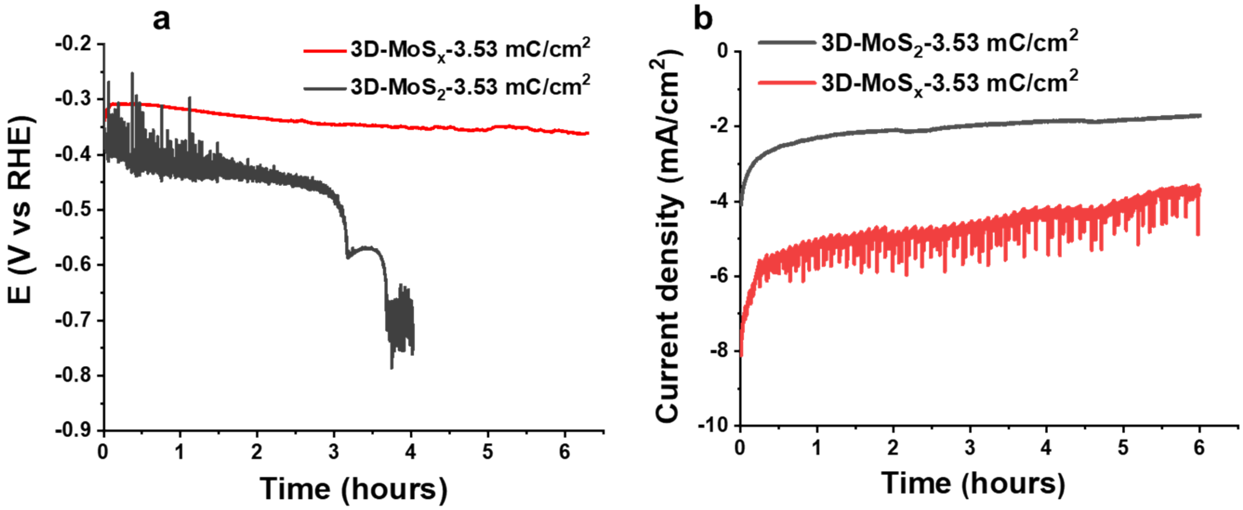

Figure S7. *(a) Stability of 3D-MoS$_x$ and 3D-MoS$_2$ catalyst electrode assayed by (a) applying a constant catalytic current density of 10 mA/cm^2 and (b) applying a constant cathodic potential of -0.3 V vs RHE for 6 hours. Electrolyte was a 0.5M H_2SO_4 solution (pH 0.3)*

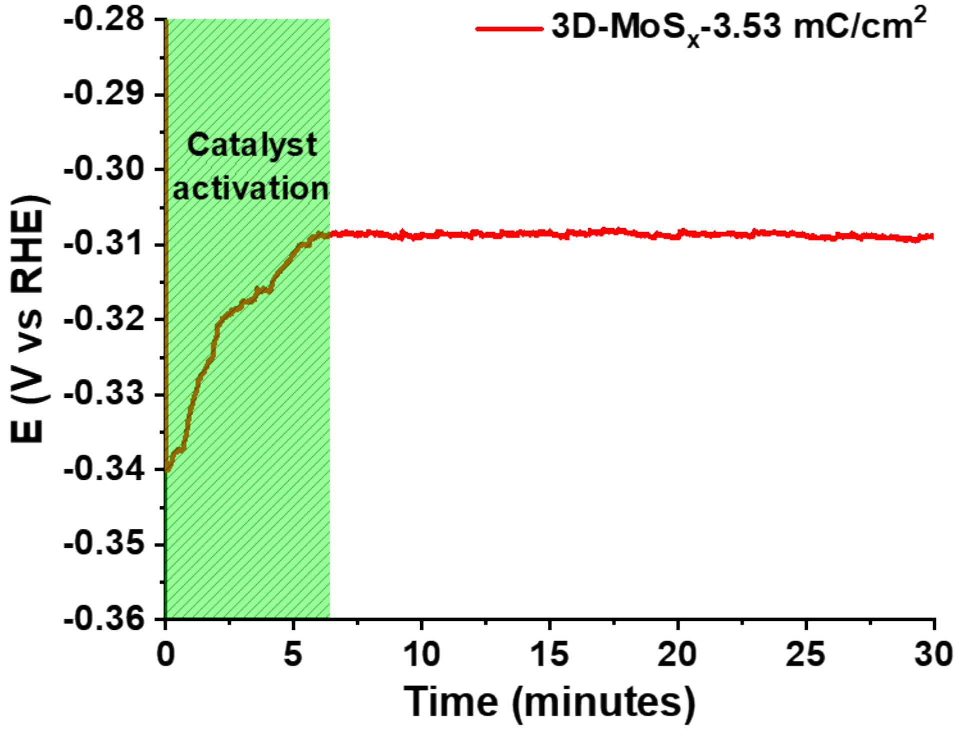

Figure S8. *E-t curve recorded on the 3D-MoS$_x$-3.53mC/cm^2 electrode conditioned at a constant catalytic current density of 10 mA/cm^2 in a 0.5M H_2SO_4 (pH 0.3) electrolyte solution*

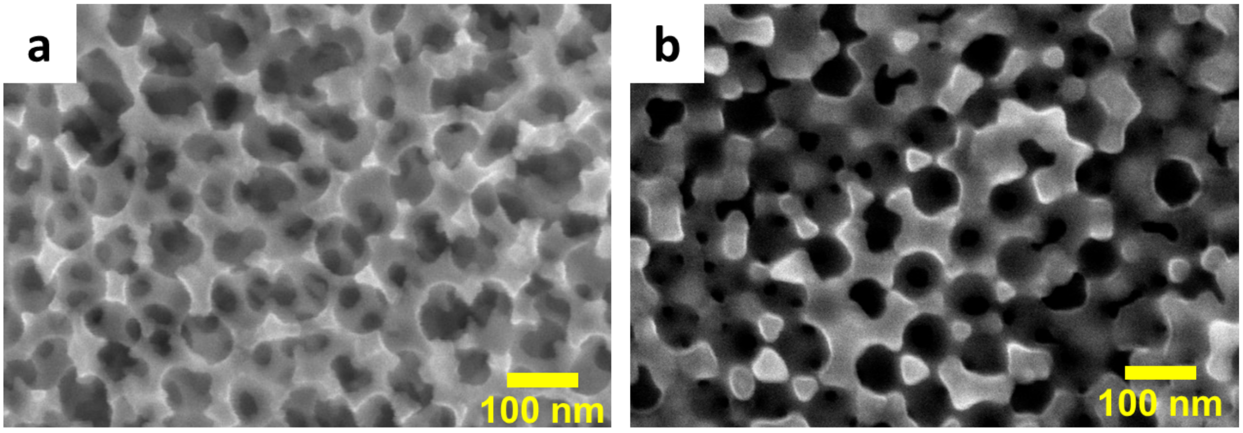

Figure S9. *Surface SEM images recorded on a (a) 3D-MoS$_2$ and (b) 3D-MoS$_x$ film after 6 hours of bulk electrolysis at -0.3V vs. RHE. Electrolyte was a 0.5M H_2SO_4 solution (pH 0.3)*

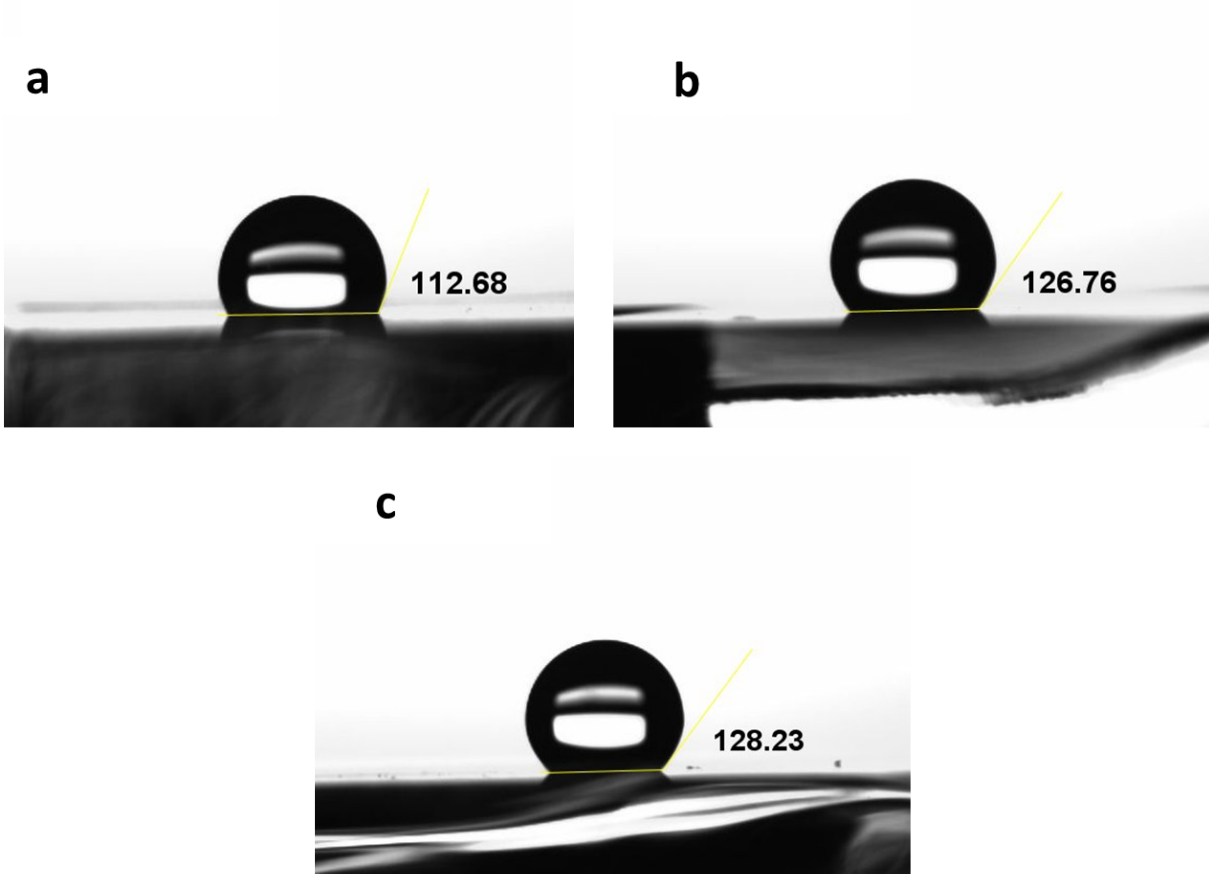

Figure S10. *Water contact angles determined for (a) a bulk* MoS_x*, (b) a 3D-*MoS_x*, and a (c) a* MoS_2 *film*

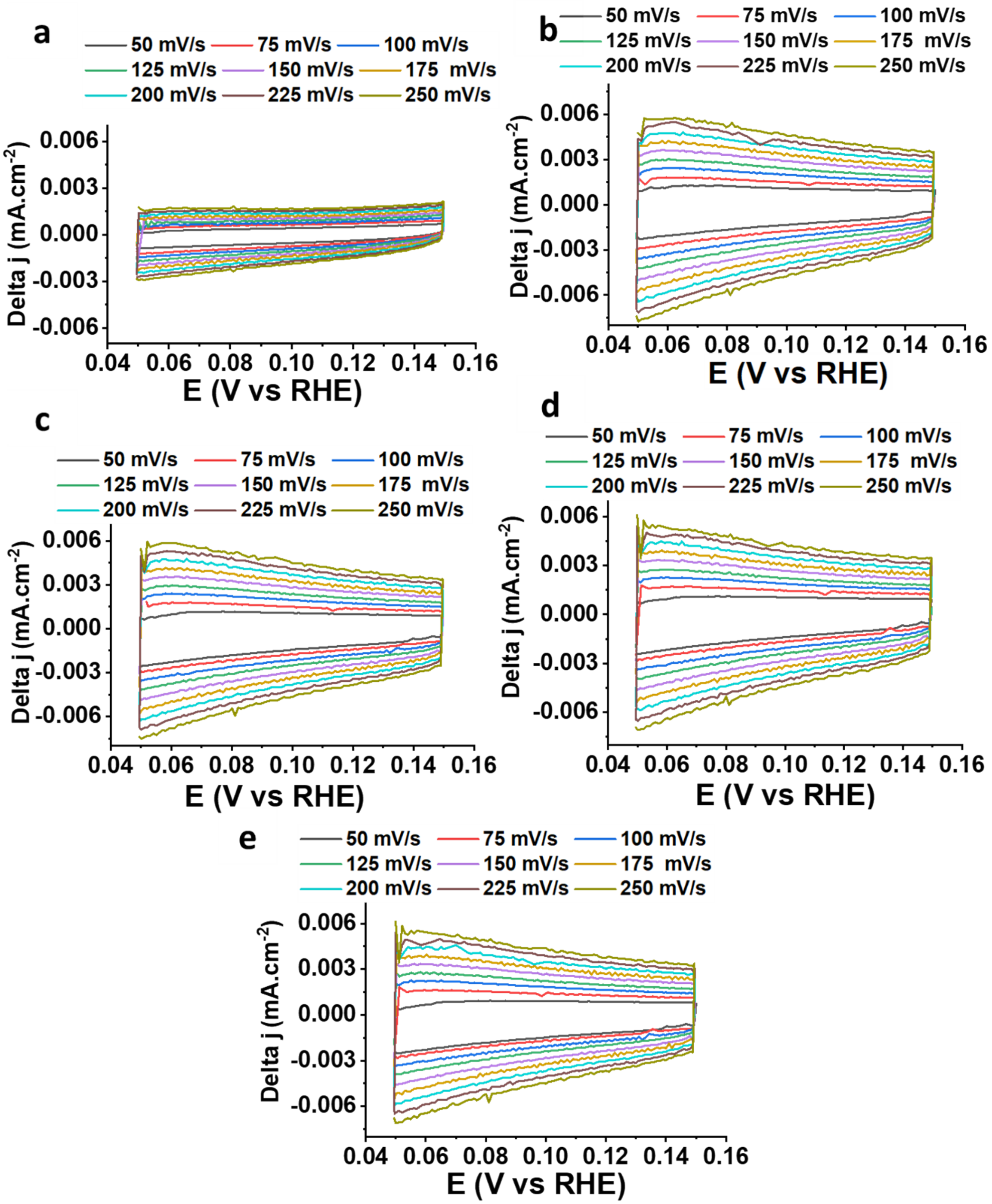

Figure S11. *Cyclic voltammograms recorded at different potential scan rates in the capacitive current region on: (a) the bulk-MoS_x-3.53 mC/cm^2, (b) 3D-MoS_x-3.53 mC/cm^2, (c) 3D-MoS_x-10.59 mC/cm^2, (d) 3D-MoS_x-17.75 mC/cm^2, (e) 3D-MoS_x-24.71 mC/cm^2. Electrolyte was a 0.5M H_2SO_4 solution (pH 0.3)*

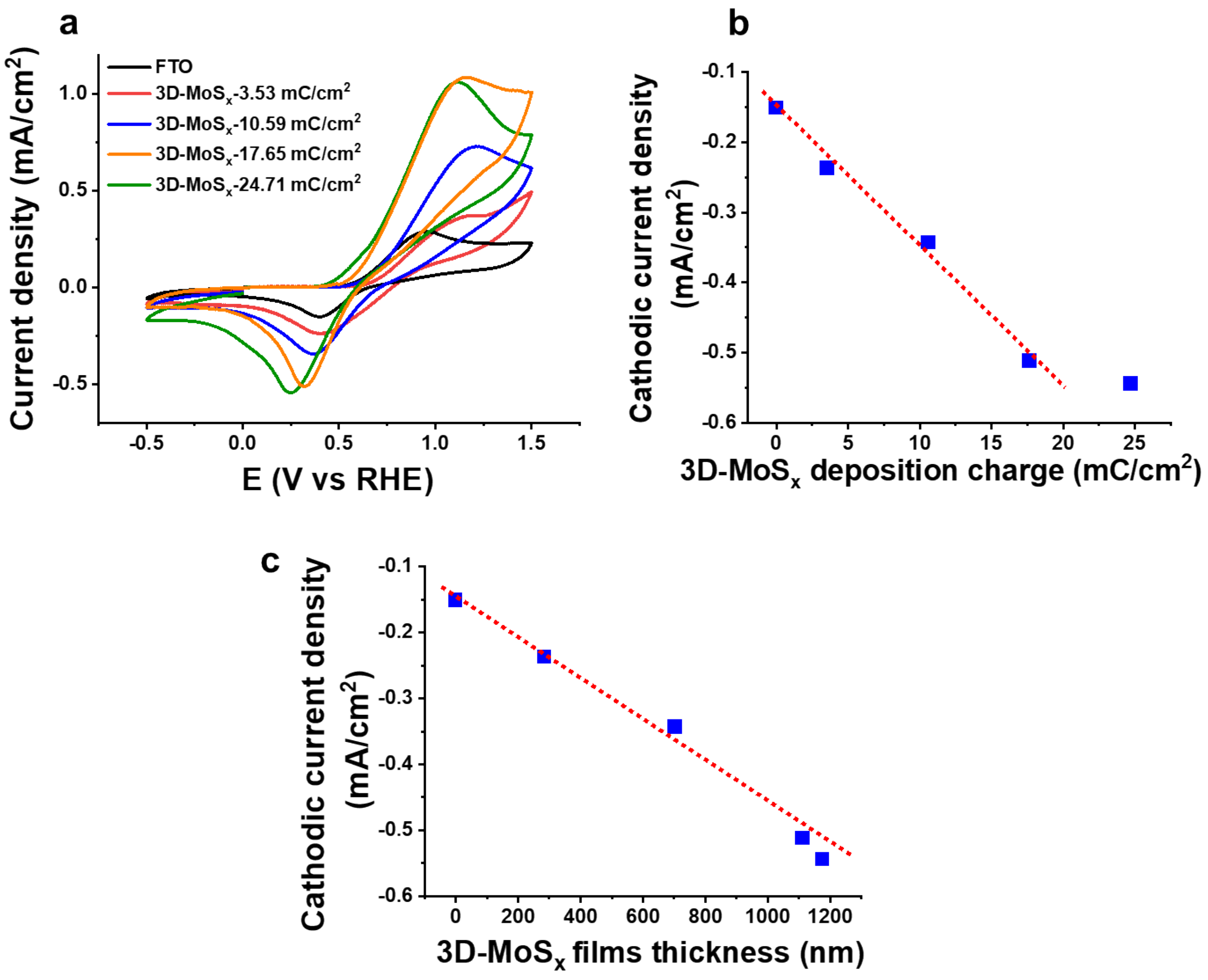

Figure S12. *(a) Cyclic voltammograms recorded for 1mM ferrocene in DCM (with 0.1M TBATFB electrolyte support) on 3D-MoS$_x$ with different thickness. Potential scan rate was 50 mV/s. (b) Plotting the cathodic current density of the Fc^+/Fc couple in function of the 3D-MoS$_x$ deposition charge density. (c) Plotting the cathodic current density of the Fc^+/Fc couple in function of the 3D-MoS$_x$ films thickness*

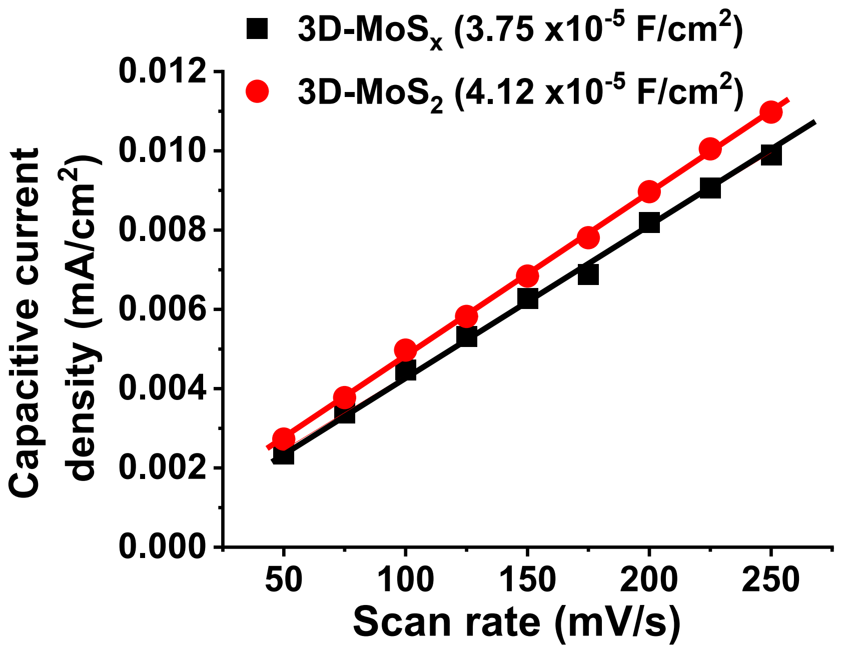

Figure S13. *Plotting capacitive current density obtained at 0.1 V vs. RHE in function of potential scan rate for 3D-MoS$_2$-3.53 mC/cm^2 and 3D-MoS$_x$-3.53 mC/cm^2 electrodes*

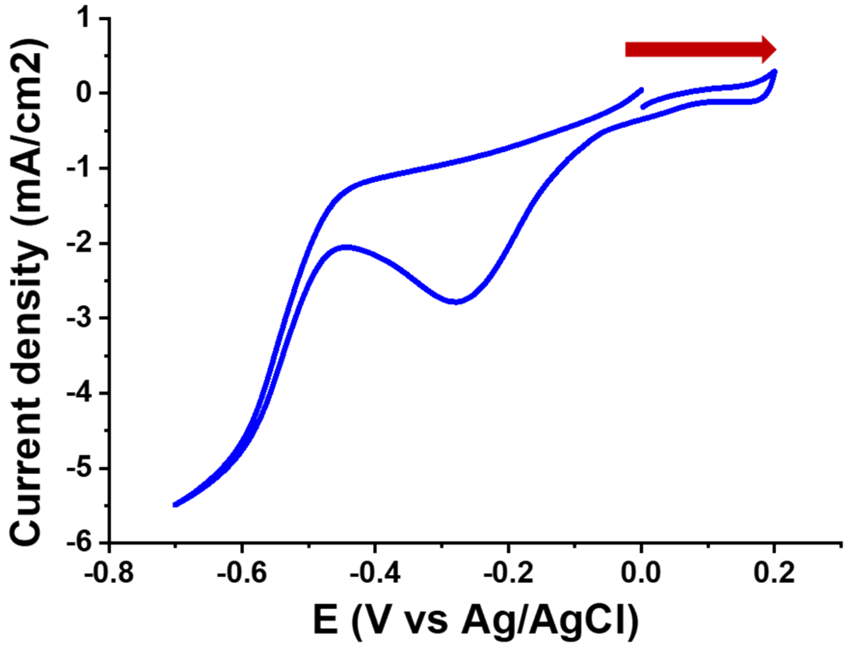

Figure S14. *Cyclic voltammogram recorded for a solution constituted of 1mM Cu_2SO_4 and 0.5M H_2SO_4 employing the 3D-MoS$_x$-10.59 mC/cm^2 electrode. Potential scan rate was 50 mV/s toward the anodic direction.*

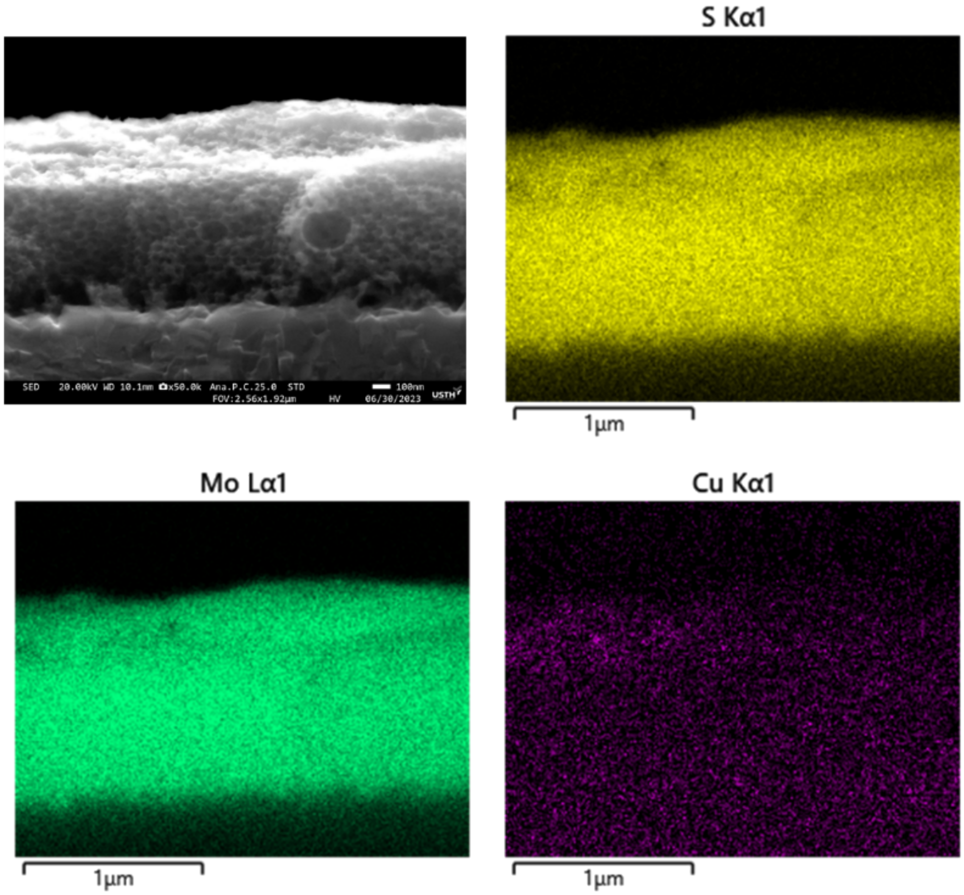

Figure S15. *EDS elemental mapping (Mo, S and Cu element) on the cross-section of 3D-*MoS_x*-10.59 mC/*cm^2 *film after being conditioned by cycling potential between -0.7 V to 0.2 V vs. Ag/AgCl with a potential scan rate of 50 mV/s. Electrolyte was constituted of 1mM* $CuSO_4$ *and 0.5M* H_2SO_4

***Calculation of theoretical surface area of 3D-*MoS_x**

Assume that the polystyrene particles are homogeneous perfect spheres and the arrangement of PS particles on FTO electrodes forms a hexagonal close-packed lattice structure (Figure S12). Each crystal unit cell contains 6 polystyrene particles.

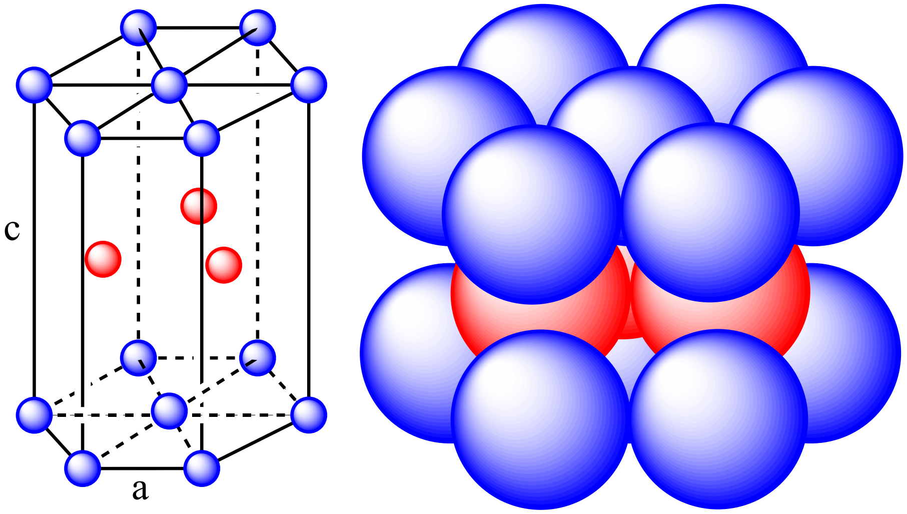

Figure S16. The hexagonal close-packed lattice structural arrangement of PS particles

+ The volume of unit cells is calculated as following: $V_{unit\ cell} = 24\sqrt{2}.R^3$ (nm^3)

+ Assume that the working electrode was prepared with a working area of 1 cm^2. The 3D-MoS_x was electrochemically deposited to the d (nm) of thickness.

The volume of 3D-MoS_x films will be calculated as: $V_{(3D\text{-}MoSx)} = (1x10^{14}).\ d$ (nm^3)

We then calculate the number of polystyrene particles in the deposited volume of 3D-MoS_x electrodes:

Number of polystyrene particles: $N = 6\,\frac{V(3D - MoSx)}{V(unit\ cell)}$

+ After removal of PS particles template, we can assume that the surface area of each polystyrene particle is equal to the remain surface area of each MoS_x inverse opal pore. Then the theoretical surface area of 3D-MoS_x can be defined:

$S_{theo.} = N\ x\ \frac{4}{3}\pi R^3$

The theoretical calculated surface values are presented in the table 2.

Table 2. Theoretical calculation of surface area on 3D-MoS_x samples with geometrical area of 1.0 cm^2

Run	Sample	Diameter of PS (cm)	Volume of unit cell (cm$^{3)}$	Thickness of deposited film (cm)	Volume of deposited film (cm^3)	Number of PS particles in film	Surface area of a PS particle (cm^2)	Theoretical surface area (cm^2)
**1**	Bulk MoS_x*	0	0	-	-	-	-	**1.000**
**2**	3D-MoS_x-35nm	3.5x10^{-6}	14.553x10^{-15}	3.0x10^{-5}	3.0x10^{-5}	12.369x10^{10}	1.539x10^{-10}	**19.041**
**3**	3D-MoS_x-50 nm	5.0x10^{-6}	42.427x10^{-15}	3.0x10^{-5}	3.0x10^{-5}	4.243x10^{10}	3.142x10^{-10}	**13.328**
**4**	3D-MoS_x-60 nm	6.0x10^{-6}	73.314x10^{-15}	3.0x10^{-5}	3.0x10^{-5}	2.455x10^{10}	4.524x10^{-10}	**11.107**
**5**	3D-MoS_x-70 nm	7.0x10^{-6}	11.642x10^{-14}	3.0x10^{-5}	3.0x10^{-5}	1.546x10^{10}	6.158x10^{-10}	**9.520**
**6**	3D-MoS_x-90 nm	9.0x10^{-6}	24.743x10^{-14}	3.0x10^{-5}	3.0x10^{-5}	7.275x10^{9}	10.179x10^{-9}	**7.405**
**7**	3D-MoS_x-90 nm	9.0x10^{-6}	24.743x10^{-14}	5.0x10^{-5}	5.0x10^{-5}	1.212x10^{10}	10.179x10^{-9}	**12.341**
**8**	3D-MoS_x-90 nm	9.0x10^{-6}	24.743x10^{-14}	7.0x10^{-5}	7.0x10^{-5}	1.697x1010	10.179x10^{-9}	**17.278**
**9**	3D-MoS_x-90 nm	9.0x10^{-6}	24.743x10^{-14}	11.0x10^{-5}	11.0x10^{-5}	2.667x10^{10}	10.179x10^{-9}	**27.151**

*The surface area of bulk MoS_x is considered as geometrical working electrodes area of 1 cm^2
